# Astrocyte remodeling without gliosis precedes optic nerve Axonopathy

**DOI:** 10.1186/s40478-018-0542-0

**Published:** 2018-05-10

**Authors:** Melissa L. Cooper, John W. Collyer, David J. Calkins

**Affiliations:** Department of Ophthalmology and Visual Sciences, The Vanderbilt Eye Institute, Vanderbilt University Medical Center, 1105 Medical Research Building IV, Nashville, TN 37232-0654 USA

**Keywords:** Neurodegeneration, Glaucoma, Glia, Astrocytes, Retinal ganglion cell, Connexin 43, Axonopathy

## Abstract

Astroyctes serve myriad functions but are especially critical in white matter tracts, where energy-demanding axons propagate action potentials great distances between neurons. Axonal dependence on astrocytes for even normal function accentuates the critical role astrocytes serve during disease. In glaucoma, the most common optic neuropathy, sensitivity to intraocular pressure (IOP) challenges RGC axons early, including degradation of anterograde transport to the superior colliculus (SC). Astrocyte remodeling presages overt axon degeneration in glaucoma and thus may present a therapeutic opportunity. Here we developed a novel metric to quantify organization of astrocyte processes in the optic nerve relative to axon degeneration in the DBA/2 J hereditary mouse model of glaucoma. In early progression, as axons expand prior to loss, astrocyte processes become more parallel with migration to the nerve’s edge without a change in overall coverage of the nerve. As axons degenerate, astrocyte parallelism diminishes with increased glial coverage and reinvasion of the nerve. In longitudinal sections through aged DBA/2 J nerve, increased astrocyte parallelism reflected elevated levels of the astrocyte gap-junction protein connexin 43 (Cx43). In the distal nerve, increased Cx43 also indicated with a higher level of intact anterograde transport from retina to SC. Our results suggest that progression of axonopathy in the optic nerve involves astrocyte remodeling in two phases. In an early phase, astrocyte processes organize in parallel, likely through gap-junction coupling, while a later phase involves deterioration of organization as glial coverage increases and axons are lost.

## Introduction

Astrocytes serve myriad functions in the mammalian central nervous system; aside from providing structural support, they additionally maintain the extracellular environment, optimize neuronal signaling, and release neurotransmitters themselves [39, 57]. Astrocytes are especially critical in white matter tracts, where axons propagate energy demanding action potentials great distances from their nuclei [29, 45]. Due to this relationship, astrocyte-axon interactions are increasingly recognized as important both in neural homeostasis and in pathology. Astrocytes exhibit multiple phases of remodeling due to neurodegenerative stress, demonstrating nuanced responsiveness and providing depth to a previously binary ‘reactive or quiescent’ classification system [33, 45, 46, 51]. Intriguingly, early astrocyte responses to neurodegeneration are often highly beneficial to injured neurons [16, 27, 32, 50]. In the optic nerve, astrocytes provide biochemical support and maintain extracellular ion balance for retinal ganglion cell (RGC) axons, which transmit retinal signals to visual structures in the brain [1, 53]. Glaucomatous optic neuropathy (or simply glaucoma), a neurodegenerative disease projected to affect some 11 million people by 2020 [37], selectively targets RGCs and their axons. Although age is the greatest risk factor, intraocular pressure (IOP) remains the only modifiable risk factor [19, 54]. However, many patients continue to lose RGC axons despite IOP-lowering treatments [26]. RGC axons and the glia that support them are particularly sensitive to age-related stressors, such as deficits in metabolism and loss of anterograde axonal transport, both of which are common aspects of age-related neurodegenerative disease [2, 5, 7–9, 40]. Recent work has focused on early phases of axonal pathology in order to abate axon susceptibility prior to overt loss [12, 35].

In neurodegenerative disease, a glial scar generally fills volume previously occupied by axons [45]. Such reactive gliosis contributes to remodeling within the optic nerve during glaucomatous progression, as in the DBA/2 J mouse model of hereditary glaucoma [3, 4, 22, 30, 43, 48]. However, earlier components of astrocyte remodeling presage overt axonal degeneration [11]. Early RGC axonopathy in animal models of glaucoma is characterized by enlargement of the optic nerve concurrent with expansion of individual axons, accumulation of hyperphosphorylated neurofilaments, and loss of anterograde transport from the retina to central brain targets [11, 13, 36]. In the DBA/2 J mouse, astrocyte processes retract from local axon bundles and redistribute from the center of the nerve to the edge before reclaiming additional coverage later in progression [11]. Early morphological remodeling may be reversible without leading to discernible axonal damage [33, 51]. Thus, understanding how astrocyte remodeling progresses early and its relationship to axonopathy may present novel therapeutic opportunities.

Here we measured defining features of astrocyte morphological remodeling that presage overt axonal degeneration in the optic nerve. We developed a novel method to quantify organization of astrocyte processes relative to their orientation during axonal degenerative progression [11, 30]. We find that astrocyte processes lose parallel orientation as axons are lost, coincident with changes in overall glial area and the distribution of astrocytes in the nerve. Diminished organization is closely related to axonal properties, rather than to changes in age or IOP. Parallelism closely relates both to astrocyte connectivity through gap junctions and maintenance of axonal anterograde transport.

## Materials and methods

### Animals and tissue preparation

The Vanderbilt University Medical Center Institutional Animal Care and Use Committee approved all animal work and experimental procedures. We obtained DBA/2 J and DBA/2 J-*Gpnmb*^*+*^*/SjJ* (‘D2 control’) mice from Jackson Laboratories (Bar Harbor, ME). A subset of DBA/2 J mice were bred in a pathogen-free facility and regularly backcrossed with fresh founders obtained from Jackson Laboratories to minimize genetic drift, as previously described [6, 21]. As described [11], 48 DBA/2 J optic nerves were used in the cross-sectional analyses within this study (Table 1). An additional 10 DBA/2 J mice and 8 D2 control mice were utilized in the immunolabeling portion of this study, all of which were harvested at 10 months. All mice were maintained on a 12 h light-dark cycle with standard rodent chow available ad libitum. We measured IOP monthly in a subset of these DBA/2 J mice representing 20 eyes using TonoPen XL rebound tonometry as previously described [11, 21]. Briefly, prior to measurement, the mice were anesthetized (Avertin, 1.3% tribromoethanol, 0.8% tert-amyl alcohol) and proparacaine ophthalmic solution (0.5% proparacaine hydrochloride, Bausch&Lomb, Tampa, FL) was applied topically to the eye. Monthly IOP for each eye was taken as the average of 25–30 Tono-Pen measurements recorded during a session.

**Table 1:** Nerves used for cross-sectional analysis

Age (mo.)	Cohort size
1.5	5
3	6
4	2
5	8
6	6
8	7
9	4
10	2
11	2
13	6

All mice were transcardially perfused with PBS followed by 4% paraformaldehyde in PBS. A 1–3 mm section of optic nerve proximal to the globe was isolated and post-fixed for 1 h in 4% paraformaldehyde. Nerves were prepared for embedding in Epon resin and semi-thin (1–2 μm) cross-sectioning as described previously [11, 14, 21, 25, 41, 42]. Cross-sections were imaged using an Olympus Provis AX70 microscope equipped with a motorized X-Y-Z stage, a digital video camera, and 100× oil-immersion, differential interference contrast optics. Photomicrographs were obtained *en montage* to represent the entire cross-section.

A subset of eyes and optic nerves from DBA/2 J and D2 control mice were enucleated and dissected from the optic chiasm for longitudinal sectioning and immunohistochemical staining. Tissue was cryoprotected in a sequence of 10, 20, and 30% sucrose/PBS overnight and subsequently embedded and frozen in Tissue-Plus O.C.T. Compound (Fisher Healthcare, Houston, TX). We labeled 10 μm cryosections with the following antibodies: anti-glial fibrillary acidic protein (GFAP; EMD Millipore, Billerica, MA, 1:500), and anti-Connexin-43 (Cx43; Alomone Labs, Jerusalem, Israel, 1:250). Immunolabeling was visualized using appropriate DyLight-conjugated secondary antibodies (Jackson Immunoresearch, West Grove, PA 1:200). Fluorescent montages were captured using an Olympus Provis AX70 microscope as described above. Confocal images were captured using an Olympus FV-1000 inverted microscope. Settings were kept constant for all sections so that comparisons in label intensity could be made.

### Nerve and axon quantification

We measured cross-sectional nerve area in a total of 110 DBA/2 J optic nerves and axon density (axons/mm^2^) in a subset of 46 DBA/2 J optic nerves as described previously [3, 6, 21]. Briefly, we randomly selected 25–30 non-overlapping frames representing a known area of nerve. Previously developed routines were used to identify and count each axon for which a single, intact myelin sheath could be identified. Nerves with pathology so severe that intact myelin sheaths were difficult for the program to detect were excluded. The mean axon density relationship for all frames was taken as the representative axon density for the nerve. In these same 46 nerves, we additionally determined the cross-sectional area for each identified axon within a myelin sheath. A range of 6000–40,000 measurements were used to calculate mean axon area (in um^2^). We calculated correlation coefficients from best-fitting regression via Pearson’s coefficient.

### Quantification of glial area and process organization

Optic nerve sections were filtered using a suite of MATLAB routines (MathWorks, Natick, MA) to generate a binary image that highlighted glial processes (described in [11]). Glial area and center of mass (CoM) were calculated as described previously. Briefly, the sum of all identified processes was used to determine the percentage of the nerve occupied by glia; the CoM was obtained by dividing the binary image into 20 concentric rings from the edge to the center of the nerve, measuring the percent glial area within each, and determining which division delineated 50% of total glial area on either side. Thus, a nerve with an even distribution of glial area between edge and center has a CoM of 10; nerves with glial area biased towards the edge have a lower CoM, while nerves with glial area biased towards the center have a higher CoM.

Binary images were additionally used to quantify glial organization by measuring the overall parallelism of the cells’ processes. The outer 20% of each nerve’s area was first eroded in order to remove the disorganized glia along the nerve edge. This erosion maximized the signal-to-noise ratio and thus the robustness of the final organization parameter. Next, the eroded image was skeletonized and pruned, reducing each binary object to a one-pixel wide collection of segments. The length of each segment was determined as the maximum with constant orientation between corners, with each corner defining a branch point to a different orientation. The branch points between segments were then removed in order to calculate a vector for each segment. The magnitude (*M*) and the directional angle (***θ***) of each organizational vector were computed using Eqs. 1–2. These values were then used to calculate the horizontal (x) and vertical (y) components of each vector (Eqs. 3–4). This process was repeated as the eroded image was rotated one degree at a time until the sum of all vertical components reached an absolute minimum, indicating the orientation of maximal horizontal orientation of glial processes in the nerve. After finding this optimal rotational angle, the final organizational parameter was derived using Eq. 5. A parallelism percentage of 100% corresponds to a theoretical nerve that is perfectly organized, while 0% describes a nerve that is perfectly disorganized. Parallelism percentages above 50% were rarely observed because glial cell bodies typically have a rounded shape, and thus the vectors derived from their skeletons are not oriented parallel to one another.

1 $$ M=\sqrt{{\left({y}_2-{y}_1\right)}^2+{\left({x}_2-{x}_1\right)}^2} $$

2 $$ \theta =\left|\arctan \left(\frac{y_2-{y}_1}{x_2-{x}_1}\right)\right|,\kern0.5em \mathrm{for}\ \mathrm{branch}\ \mathrm{endpoints}\ \left({x}_1,{y}_1\right),\left({x}_2,{y}_2\right) $$

3 $$ x\_ comp=M\cos\ \theta $$

4 $$ y\_ comp=M\sin\ \theta, \kern0.75em \mathrm{where}\ \theta \in \left[0,90\right] $$

5 $$ Parallelism=\left(1-\frac{\min {\sum}_{i=1}^ny\_{comp}_i}{\max {\sum}_{i=1}^nx\_{comp}_i}\right)\times 100 $$

### Tracing of anterograde axonal transport

Forty-eight hours prior to perfusion, a subset of animals were anesthetized with 2.5% isoflurane and bilaterally injected intravitreally with 2 μl of 0.5 mg cholera toxin subunit B (CTB) conjugated to Alexa Fluor 488 (Invitrogen) as previously described [13, 14]. Two days post-injection, animals were transcardially perfused with PBS followed by 4% paraformaldehyde in PBS. Brains were cryoprotected in 30% sucrose/PBS overnight, and 50 μm coronal midbrain sections were cut on a freezing sliding microtome. Serial superior colliculus sections were imaged using a Nikon Eclipse TI microscope (Nikon Instruments) and the intensity of the fluorescent CTB signal was quantified using ImagePro custom routines (Media Cybernetics) as previously described [13, 56]. CTB signal was normalized to background and alternating sections were analyzed for intensity. Intensity from each section was calculated to reconstruct a retinotopic map of intact anterograde transport across the superior colliculus. Intact transport for each map was defined as any region with an intensity ≥ 70% of the maximum CTB signal for that tissue.

### Statistical analysis

Data for IOP and glial area analysis are presented as mean ± standard error of the mean (SEM) for each treatment. Statistical analysis and *p*-values for comparing means were obtained using Kruskal-Wallis one-way ANOVA or two-sided t-tests, and all data met criteria for normalcy as confirmed using the Shapiro-Wilk normality test; all datasets compared passed with *p* ≥ 0.22. Correlations were calculated using the two-tailed Pearson product moment test and verified by linear regression analysis. Statistical tests were considered significant if *p* < 0.05. All statistical tests were performed with SigmaPlot 12.5 (Systat Software Inc., San Jose, CA). Numbers of samples and measurements along with actual *p* values of significance are indicated where appropriate in the text or figure legends.

## Results

### Optic nerve remodeling involves diminished astrocyte organization

**Fig. 1:**
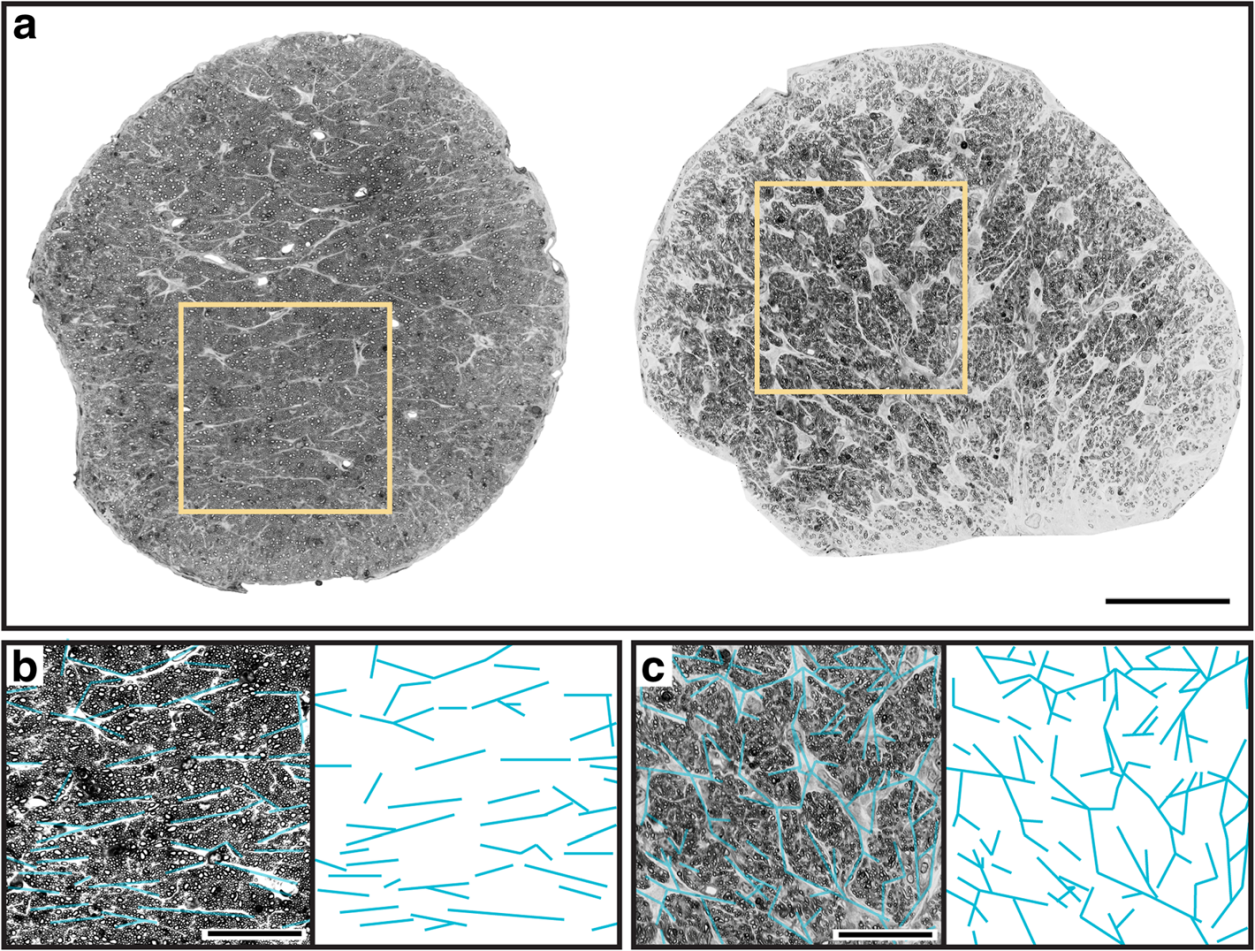
Pathology alters glial orientation in the optic nerve. **a** Left: cross-section through DBA/2 J optic nerve prior to progression. Nerve has high axon packing density (6.2 × 10^5^ axons/mm^2^) and low glial coverage area (11.4% of total nerve area). Right: during progression axons are lost (2.4 × 10^5^ axons/mm^2^) and glial coverage increases (45.19%). **b** Higher magnification of inset from healthy nerve in **a** with highlighted glial processes demonstrating parallel orientation. **c** Inset from glaucomatous nerve in **a** with glial processes similarly highlighted. In this nerve, processes appear disordered, or oriented without a discernable pattern. Scale = 100 μm (**a**), 10 μm (**b**, **c**)

Astrocyte processes retract from axon bundles prior to frank loss of axons in the myelinated segment of the DBA/2 J optic nerve [11]. Using the same cohort of nerves, here we report another interesting characteristic of astrocyte organization and orientation. Fig. 1a (left) shows a healthy nerve with high axon density (6.2 × 10^5^ axons/mm^2^) and a low fraction of its area covered by glia (11.4%). For this nerve, astrocyte processes appear to follow a parallel pattern of distribution (Fig. 1b). This pattern is consistent with astrocyte distribution in cross-section of healthy white matter tracts [28, 31]. However, a glaucomatous nerve with substantial loss of axons (2.4 × 10^5^ axons/mm^2^) and increased glial coverage (45.19%) demonstrates clear disorganization of astrocyte processes (Fig. 1a, right), with less apparent parallel orientation (Fig. 1c).

**Fig. 2:**
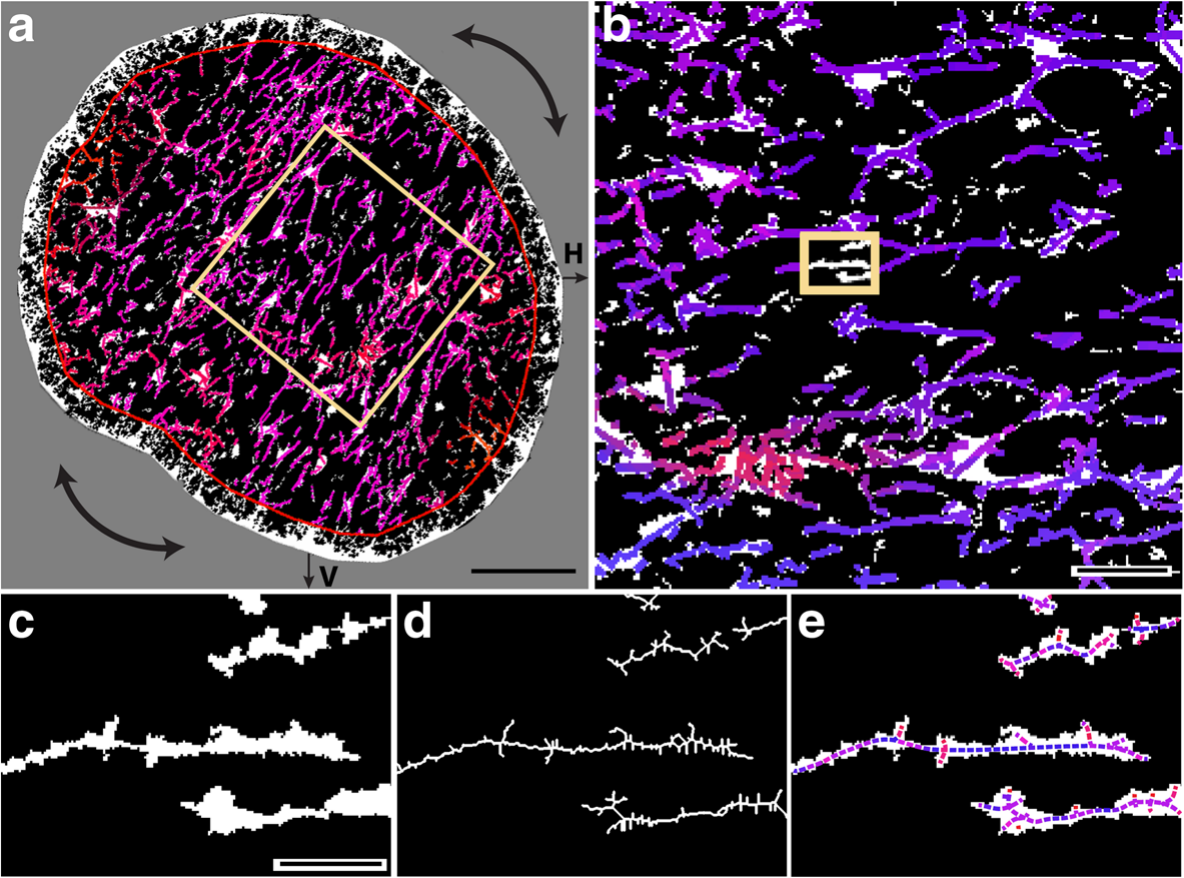
Deconstruction of astrocyte processes into representative vectors. **a** Binary representation of an 8-month DBA/2 J nerve with glial processes highlighted to indicate coverage (white; 19.35% of nerve area). Colored overlay demonstrates result of algorithm to calculate vectors, with degree of orientation along the vertical axis (V) indicated by red tint and degree of orientation along the horizontal axis (H) indicated with blue tint. Our algorithm excludes the outer 20% of nerve area to minimize edge effects and rotates the image to maximize orientation along the horizontal axis, shown by the inset magnified in panel **b**. c Inset from **b** in higher magnification shows glial processes in binary image. **d** Same glial processes skeletonized for assigning vectors to segments of constant orientation. **e** Representative resultant vectors for each segment calculated from skeletonized image overlaid on original binary representation of glial processes. The length of each vector represents the pixel distance of each segment between branch points in the skeletonized image. Side branches less than threshold length or width were excluded (see Materials and methods). Scale = 100 μm (**a**), 10 μm (**b**), 2 μm (**c**, **d**, **e**)

To quantify loss of parallelism in our cross-sections of proximal DBA/2 J optic nerve, we devised an algorithm that decomposes glial processes into horizontal and vertical vector components. Using a binary representation of each nerve in which glial processes are highlighted (Fig. 2a; see Fig. 7 of [11]), each nerve was rotated to maximize the orientation of glial processes along the horizontal axis (Fig. 2b). This normalizes images for nerve orientation. Following rotation (Fig. 2c), each process was skeletonized to simplify decomposition into vectors (Fig. 2d). Skeletons were decomposed by removing branch points, resulting in a population of vectors representing the orientation of each segment of astrocyte processes (Fig. 2e).

**Fig. 3:**
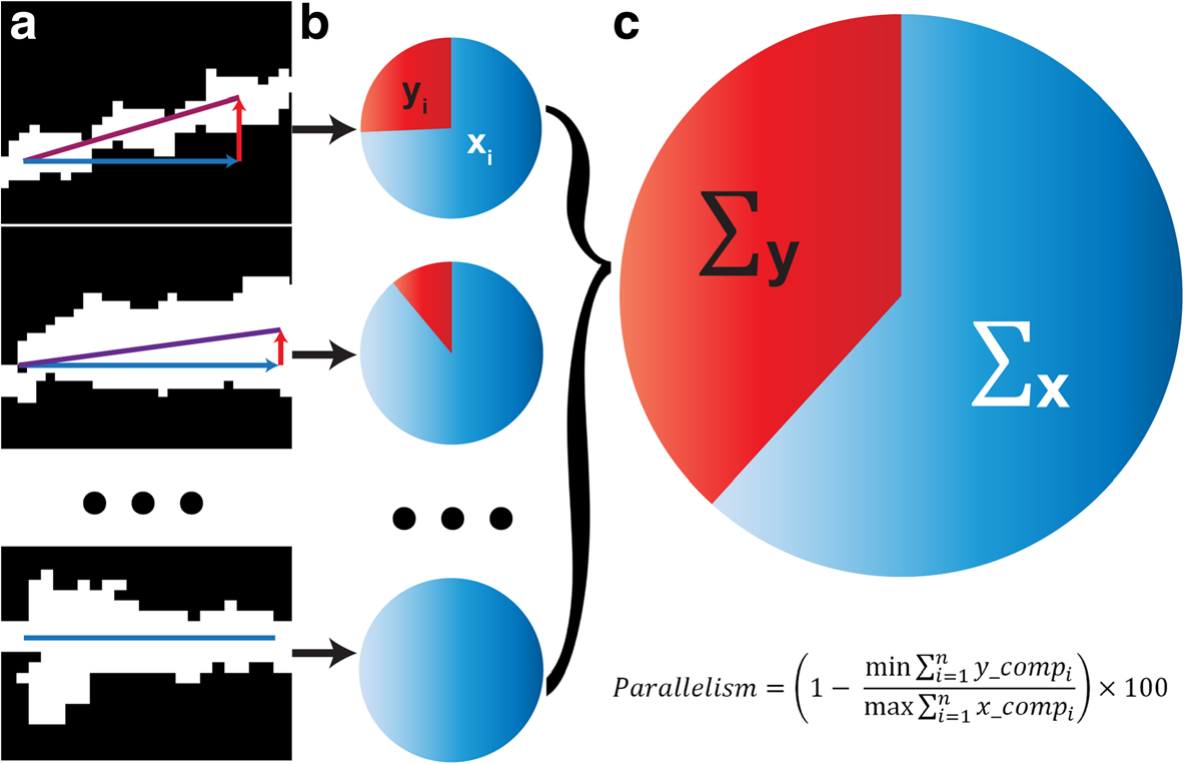
Method to quantify the overall parallelism of glial processes. **a** Binary high-magnification images of glial processes with increasing horizontal vector components (blue) compared to vertical (red). Resultant vector is the sum of these components (purple). **b** Individual pie charts depict the ratio of the length of the horizontal (blue, x_i_) and vertical (red, y_i_) components for each resultant vector. **c** The sum of the horizontal (x) and vertical (y) components for each nerve represents the degree of parallelism. The percent parallelism for each nerve is calculated from the ratio of the summed components following rotation to minimize vertical and maximize horizontal orientation. 100% corresponds to perfect parallelism and 0% corresponds to random orientation

By collecting all such segments across the nerve, we generated a single representative value of astrocyte parallelism. For the collection of vectors (Fig. 3a), we summed the pixel length of all horizontal and vertical components (Fig. 3b). This summation defined a resultant vector representing the orientation and length of all vectors across the cross section (Fig. 3c). We defined the percent parallelism in each nerve using the ratio of summed horizontal and vertical components such that that a value of 100 indicates perfectly parallel orientation (i.e., no vertical component), while a value of 0 indicates random orientation due to equivalent vertical and horizontal components. This single value represents the organization across an entire nerve, providing a mechanism to analyze process organization in relation to other outcome measures.

**Fig. 4:**
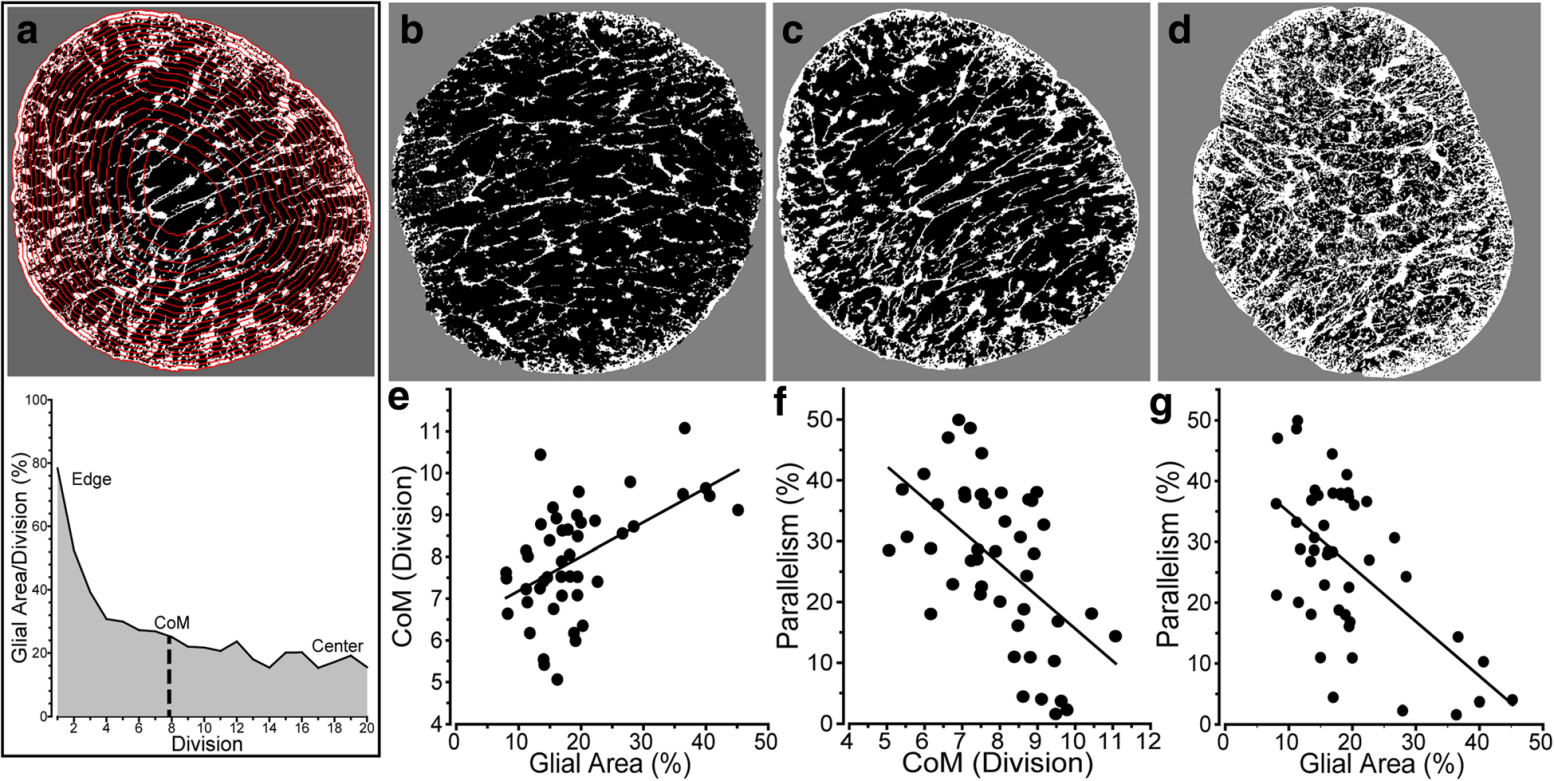
Measuring the spatial distribution of astrocyte processes. **a** Modified from Cooper et al. [11]. Center of Mass (CoM) was calculated using a series of concentric circles (red) each delineating 5% of nerve area. From the first division at the edge of the nerve to the twentieth division at the center (graph below), the CoM represents the division at which half of the glial area resides on each side. **b** Cross-section through a young (1.5 month) DBA/2 J optic nerve shows slight bias of astrocyte center of mass (CoM) towards edge (CoM = 7.22), low coverage (11.2%), and high parallelism (48.6%). **c** A 3 month DBA/2 J nerve with higher CoM (8.55) and coverage (26.7%) shows less parallelism (30.6%). **d** A 5 month nerve with expansive glial coverage (40.6%) shows even astrocyte distribution (CoM = 9.44) and loss of parallelism (10.3%). **e** CoM increases significantly with glial area (modified from [11]; *r* = 0.529, *p* < 0.001). **f**, **g** Parallelism decreases with both increasing CoM (*r* = − 0.55, *p* < 0.001) and expanding glial area (*r* = − 0.60, *p* < 0.001). Scale = 100 μm (A-C)

Astrocyte processes in the DBA/2 J optic nerve redistribute spatially during progression, showing a tendency to retreat towards the edge prior to frank axon degeneration [11]. With progression, astrocyte processes once again fill in across the nerve. In Cooper et al. [11], we quantified this trend by defining a center of mass (CoM) based on division of the nerve into concentric rings, with values near 10 indicating an even distribution and values < 10 indicating bias towards the edge of the nerve (Fig. 4a). As glial area increases, the pattern of process distribution alters (Fig. 4b, c, d). We found that nerves with lower glial area tended to have an edge-distributed CoM (about 7), while nerves with higher glial area have a relatively even glial distribution (about 10; Fig. 4e). As CoM increased and glial coverage expanded, parallelism diminished as well (Fig. 4f,g).

### Parallelism reflects early axonal changes

**Fig. 5:**
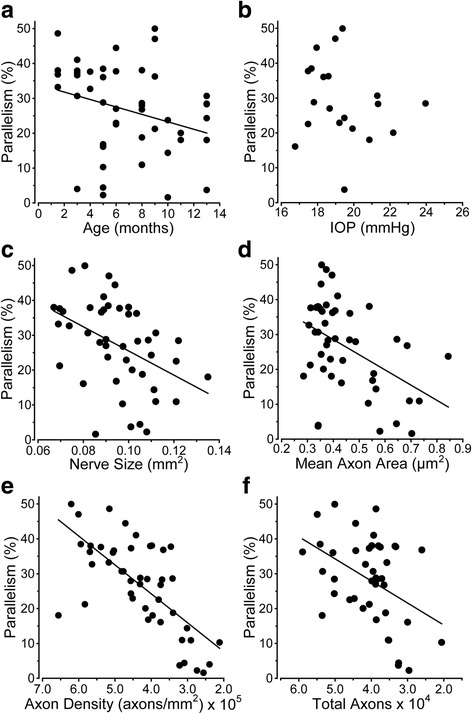
Parallelism diminishes with markers of axon degeneration. **a** Parallelism decreases moderately as DBA/2 J mice age (*r* = − 0.293, *p* = 0.046); there is no correlation with the mean lifetime IOP (**b**; *r* = − 0.193, *p* = 0.415). **c** Parallelism rapidly diminishes with enlargement of the nerve (*r* = − 0.435, *p* = 0.003). **d** Increased mean axon area is also correlated with decreased parallelism (*r* = − 0.457, *p* = 0.001). **e** Parallelism demonstrates a tight correlation with diminished axon density (*r* = − 0.628, *p* < 0.001). **f** As the total number of axons decreases, parallelism decreases (*r* = − 0.427, *p* = 0.005)

Next we examined how redistribution of astrocyte processes in the nerve is influenced by other predictors and indices of glaucomatous progression in the DBA/2 J nerves. Across our sample of optic nerves, we found that parallelism diminished only modestly with age (Fig. 5a); the relationship with IOP was even weaker (Fig. 5b). Cross-sectional nerve area (measured in mm^2^) increases with age in the DBA/2 J mouse; this is linked eventually to axon loss and significant enlargement of cross-sectional area of surviving axons [11]. Here, we found that as the nerve enlarges, parallelism decreases significantly (Fig. 5c). Accordingly, since nerve expansion predicts both axon loss and axonal enlargement [11], increased axon size (Fig. 5d), diminished axon density (Fig. 5e), and loss of total number of axons all correlated with decreased parallelism (Fig. 5f). There is, however, a caveat to this conclusion.

**Fig. 6:**
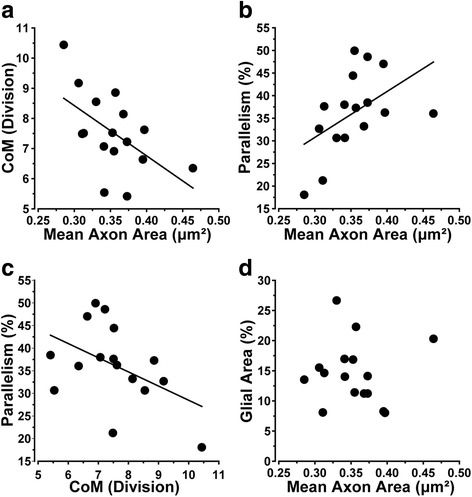
Astrocytes become more parallel as axons expand early prior to loss. **a** Modified from Cooper et al. [11]. CoM decreases significantly as axons expand early in the DBA/2 J nerve prior to overt loss of axons (*r* = 0.55, *p* < 0.001). **b** As axons expand prior to outright loss, parallelism increases (*r* = 0.50, *p* = 0.05). **c** Parallelism tends to increase with decreasing CoM for the same set of nerves (*r* = 0.47; *p* = 0.071). **d** Glial coverage of the nerve does not change as axons expand prior to loss (*r* = 0.006, *p* = 0.98). All data collected from subset of nerves with highest axon sampling density below the threshold of axon size for which loss occurs (4.8–6.5 × 10^5^ axons/mm^2^; see [11])

While axons expand in area continuously from early to late progression in the DBA/2 J optic nerve, expansion is not associated with axon loss up to a critical threshold of about 0.50 μm^2^ [11]. This early axon expansion is coincident with retraction of astrocyte processes towards the nerve edge and a temporary decrease in CoM (Fig. 6a, modified from [11]). For these same pre-degenerative nerves, we then examined parallelism. As axons expanded and astrocyte processes retracted towards the edge, parallelism increased (Fig. 6b). Intriguingly, these nerves at early stages of pathology exhibit glial distributions opposite that of the sample of nerves across all stages of degeneration (Fig. 6d). Moreover, the dependence of parallelism on CoM was slightly weaker for these pre-degenerative nerves than for the entire sample (*p* = 0.071; Fig. 6c*,* compare to Fig. 5e). For this subset of nerves, glial coverage did not change with axon size (Fig. 6d) or with parallelism (data not shown; *r* = 0.0942; *p* = 0.0729). These data indicate that while parallelism diminishes as axons degenerate later (Fig. 5e), early changes in the nerve prior to overt axon loss are associated with increased parallelism without a change in overall glial coverage.

### Astrocyte cytoskeletal reorganization depends on location in the nerve

**Fig. 7:**
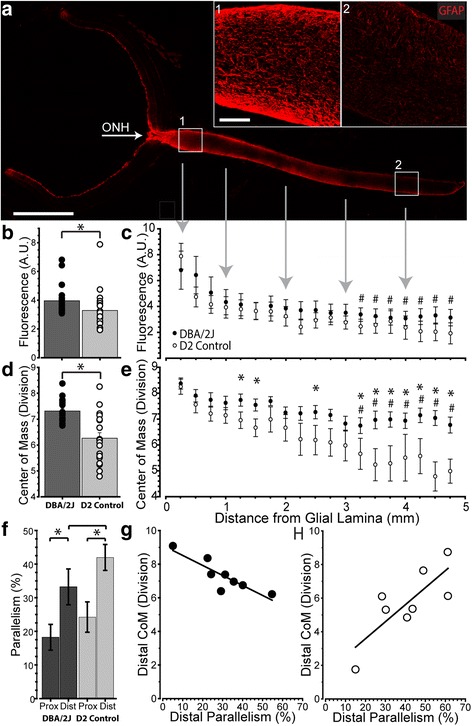
Astrocyte GFAP redistributes throughout the optic nerve. **a**. Longitudinal section through 10-month DBA/2 J retina and optic nerve shows astrocytes labeled for GFAP (red). GFAP expression is highest at the optic nerve head (ONH), proximal optic nerve (1), and at the edges of the distal nerve (2). **b**. GFAP is significantly elevated in DBA/2 J nerves compared to D2 control nerves (*p* < 0.001). **c**. When analyzed in 0.25 mm segments, no one segment of DBA/2 J nerve contains significantly elevated GFAP compared to the same D2 control segment; however, the most proximal segment (0.25 mm) in both DBA/2 J and D2 control nerves contains significantly more GFAP than distal segments (#; *p* < 0.001). **d**. The CoM of GFAP within DBA/2 J nerves is significantly greater than D2 control nerves (*p* < 0.001). **e**. DBA/2 J nerves contain significantly altered GFAP CoM from D2 control nerves, and these differences occur most often within distal nerve segments (*; *p* < 0.029). **f**. GFAP parallelism is significantly greater in the distal compared to the proximal nerve in both the DBA/2 J (*p* = 0.015) and D2 control (*p* = 0.003). Additionally, GFAP within distal DBA/2 J nerves trends toward less parallelism than the equivalent segment of D2 control nerves (*p* = 0.09). **g**. DBA/2 J distal nerves tend to have a more edge-distributed center of mass when parallelism is greater (*p* = 0.005), indicating parallelism and center of mass are related. **h**. This relationship is trending toward the reverse in distal D2 control nerves (*p* = 0.07). Scale: 1 mm (A), 100 μm (A insets)

To determine how astrocyte reorganization reflects cytoskeletal changes, we measured the distribution of GFAP in discrete intervals along longitudinal sections through the aged (10 month) DBA/2 J optic nerve, spanning proximal to the nerve head (< 1 mm) and to more distal (> 4 mm; Fig. 7a insets 1 and 2). We then compared this distribution to CoM and parallelism redefined for immuno-labeling in longitudinal sections (see Materials and methods). On average, DBA/2 J nerves exhibited increased total nerve GFAP compared to the age-matched D2 control strain (*p* < 0.001; Fig. 7b). While at discrete points along the nerve GFAP in DBA/2 J was systematically higher, this trend did not translate to a significant difference within any one interval (Fig. 7c). The CoM for GFAP was significantly elevated in DBA/2 J compared to D2 nerves, indicating a more central distribution of astrocytes (*p* < 0.001; Fig. 7d). Moreover, unlike GFAP levels, CoM did significantly depend on location in the nerve (Fig. 7e), with GFAP-labeled processes distributing more towards the nerve center at distal locations compared to D2 nerves. This is a later hallmark of progression [11]. Additionally, we found that GFAP parallelism is elevated in the distal nerve of both DBA/2 J (*p* = 0.015) and D2 control compared to proximal nerve (*p* = 0.003; Fig. 7f). Finally, the CoM for distal segments of DBA/2 J nerve indicated edge-distributed astrocytes with increased parallelism (*p* = 0.005), while CoM in D2 nerves showed the opposite trend (Fig. 7g), though not significant (*p* = 0.07).

### Parallelism reflects both astrocyte connectivity and RGC axonal function

**Fig. 8:**
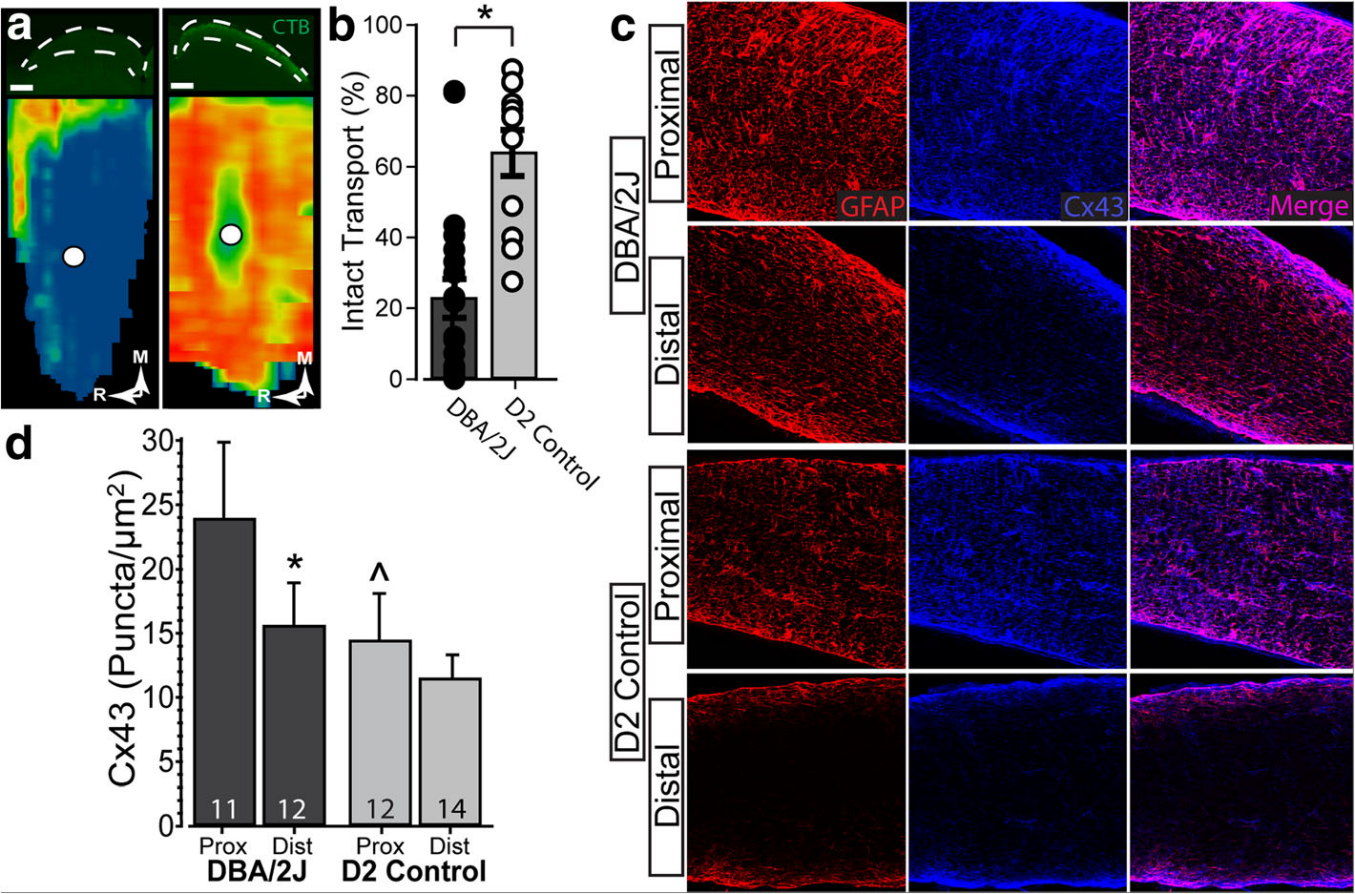
Diminished anterograde transport in a sample of DBA/2 J nerves. **a** Top Left: Coronal section through superior colliculus of DBA/2 J mouse (between dashed white lines) following intravitreal injection of CTB (green). Bottom Left: Corresponding retinotopic map shows nearly depleted anterograde transport of CTB. Top Right: Coronal section through superior colliculus of D2 control mouse prepared as on left. Bottom Right: Corresponding retinotopic map shows a full complement of anterogradely transported CTB. **b** Transport of CTB from DBA/2 J eyes was near 20% (22.8 ± 5.5), significantly reduced from D2 control which is near 75% (74.896 ± 4.328) (*p* = 0.0015). **c** Confocal micrographs of proximal (left) and distal (right) DBA/2 J (top) and D2 control (bottom) optic nerves. Connexin 43 (Cx43, blue) and GFAP (red) colocalize, and both are elevated in proximal optic nerve. **d**. Density of Cx43 (puncta/μm^2^) in proximal segment of DBA/2 J nerves is significantly elevated compared to both distal DBA/2 J (*; *p* = 0.043) and proximal D2 nerves (#; *p* = 0.032)

Loss of anterograde axonal transport of cholera toxin B (CTB) from retina to superior colliculus (SC) is an early hallmark of axonopathy in the DBA/2 J and other models [13, 18, 25, 34, 51, 56]. Consistent with this pattern, while SC from D2 animals demonstrated mostly intact transport (75% intact), DBA/2 J SC exhibited severe deficits (Fig. 8a), with intact transport averaging only about 20% (22.784 ± 5.493), a highly significant difference (*p* = 0.0015, Fig. 8b). Astrocytes couple to one another through gap junctions to form a dense network that is tightly modulated by axonal function and neuronal activity [38]. In these same animals, we examined levels of connexin 43 (Cx43), a marker for gap junctions between astrocytes [24]. Consistent with this role, both proximal and distal segments of DBA/2 J and D2 optic nerve show strong Cx43 colocalization with GFAP (Fig. 8c). Additionally, there are significantly more Cx43 puncta in proximal vs. distal segments in the DBA/2 J nerve (*p* = 0.043; Fig. 8d), similar to the distribution of GFAP (Fig. 7c). Additionally, Cx43 puncta are increased in DBA/2 J nerve compared to D2, though this is only significant for proximal segments (*p* = 0.032; Fig. 8d).

**Fig. 9:**
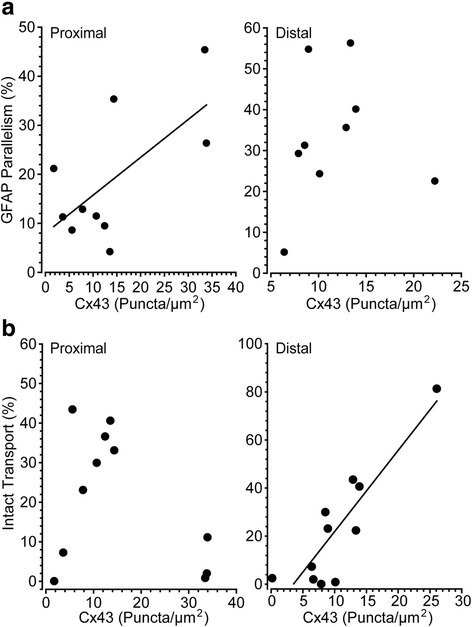
RGC axon functional deficits are associated with astrocyte connectivity in DBA/2 J optic nerve. **a**. Higher levels of Cx43 are associated with a greater degree of parallelism of GFAP-labeled astrocyte processes in proximal segments of DBA/2 J nerve (left, *R* = 0.66; *p* = 0.04) but not distal (right, *R* = 0.11; *p* = 0.77). **b** Intact anterograde transport to the superior colliculus is well-predicted by levels of Cx43 in the distal optic nerve (right, *R* = 0.88; *p* < 0.001), but not proximal (left, *R* = 0.39; *p* = 0.23). Scale: 100 μm (**a**)

Higher Cx43 in the proximal segment predicted a greater degree of parallelism for GFAP-labeled astrocyte processes (*p* = 0.04; Fig. 9a, left). This was not so for the distal segment (*p* = 0.77; Fig. 9a, right), though overall parallelism was higher in this location (Fig. 7f). These differences may have functional significance for axons. Earlier we demonstrated that anterograde transport from retina to the SC in the DBA/2 J degrades in a distal (brain) to proximal (retina) pattern [13]. Here, while Cx43 in the proximal segment did not correlate with levels of intact axonal anterograde transport (Fig. 9b, left), increased Cx43 in the distal segment strongly correlated with this measure of axonal function (*p* < 0.001; Fig. 9b, right).

## Discussion

Our results demonstrate several key features of astrocyte organization and its alterations through pathology. In cross-sections of healthy DBA/2 J optic nerve (Fig. 1), astrocyte processes organize in a similar direction, i.e., exhibit a high degree of parallelism as indicated by our metric (Figs. 2 and 3). As axon pathology increases with a commensurate increase in glial coverage of the nerve, parallelism diminishes and astrocytes distribute more evenly across the nerve (Fig. 4). Accordingly, axon density, total number of axons, and axon expansion all predict changes in astrocyte parallelism more accurately than independent measures of glaucoma progression in the DBA/2 J (i.e., IOP and age; Fig. 5). Earlier we reported that axons expand in mean cross-sectional area up to a threshold of about 0.5 mm^2^ before their number begins to decline [11]. Concomitantly, astrocyte processes retreat towards the edge of the nerve, meaning center-of-mass (or CoM) diminishes. In these pre-degenerative nerves, as mean axon area increases towards the threshold for loss, astrocyte parallelism increases without a change in overall glial coverage of the nerve (Fig. 6). Once axon loss begins and density diminishes, parallelism decreases in kind (Fig. 5e), presumably as astrocyte processes re-invade axon bundles [51]. In total, these results from the DBA/2 J nerve indicate that astrocytes remodel biphasically. During early axon expansion prior to axonal loss, astrocyte processes increase in parallelism as CoM diminishes *independent from gliosis* (Fig. 6). Once axons are lost, gliosis involves diminished parallelism and uniform astrocyte distribution across the nerve (Fig. 4).

In longitudinal sections of nerve, we found that both GFAP and Cx43 - which marks astrocyte gap junctions [24] – are higher in proximal vs. distal segments (Figs. 7c, 8d). This is consistent with a more even distribution of astrocytes, as indicated by the higher CoM in the proximal vs. the distal segment (Fig. 7e). In earlier work, we demonstrated that anterograde axonal transport from retina to brain is an early harbinger of axon pathology in the DBA/2 J and degrades in distal-to-proximal fashion [13]. We found here that parallelism of GFAP-labeled astrocyte processes was higher in distal segments of our 10-month old DBA/2 J nerves (Fig. 7f). As parallelism increased in the distal segment, astrocytes redistributed towards the nerve edge, as indicated by a lower CoM (Fig. 7g). This pattern of remodeling reflects that seen in early stages of axonal pathology, indicating that astrocytes in the distal nerve may be reacting to pathology prior to those in the proximal nerve.

In the distal segment, nerves containing elevated Cx43 also demonstrated higher levels of intact transport, but did not show a relationship with parallelism (Fig. 9b). In contrast, levels of Cx43 in the proximal segment predicted parallelism with no clear relationship to transport (Fig. 9a). Thus, these results indicate that in the distal segment, where axonopathy has already begun [13], Cx43 in astrocytes better reflects axon function than astrocyte organization. This is consistent with findings showing that gap-junction coupling of the astrocyte network is tightly modulated by axonal function and neuronal activity [38]. Similarly, increased parallelism in the distal vs. proximal segment is reminiscent of our results from cross-sections of proximal nerve, where increased parallelism correlated with early expansion of axons prior to overt loss (Fig. 6b). Through gap-junction coupling, astrocytes may be able to more evenly distribute resources to those axons undergoing the highest degree of stress [17, 20, 36, 47].

Our results suggest that early progression in the optic nerve involves remodeling of astrocyte processes, first to a higher state of organization with increased parallelism (Fig. 6) and gap-junction coupling (Fig. 9) and then to gradual deterioration of organization as coverage increases and axons are lost (Figs. 4 and 5). Perhaps well-organized astrocyte processes lend structural stability to the nerve. In the optic nerve head, astrocytes form a continuously remodeling network that adapts to IOP exposure [44], with astrocyte actin and tubulin filaments gradually re-orienting as IOP increases [52]. With short exposures to elevated IOP, astrocyte processes retract towards the cell body [51]; astrocyte processes also may also fortify the edge of the nerve, as coverage in the center diminishes (Fig. 3 of Sun et al., 2013). As astrocyte processes re-invade the nerve center with longer IOP exposure, their organization diminishes [51]. With acute injury (nerve crush), astrocyte processes appear to detach from the edge [49], but this likely reflects the more severe nature of the injury. Similar to our results in the myelinated nerve, astrocyte remodeling following induced short-term elevations in pressure is most extensive distal from the sclera [55]. Portions of the optic nerve beyond the sclera itself are unlikely to be impacted directly by elevated IOP, and secondary changes could involve different structural mechanisms. This may be why we did not note a simple linear relationship between IOP and loss of parallelism (Fig. 5b), but did find strong correlations between measures of axonal degeneration and loss of parallelism (Figs. 5 and 6).

Perhaps the most important question is whether early astrocyte remodeling is protective or pathogenic. Cx43 is elevated in the optic nerve head and the retina in human glaucomatous tissue [23], and here we find that astrocyte remodeling is associated with increased Cx43 (Fig. 9). Intriguingly, we additionally find that elevated Cx43 is also associated with intact axonal anterograde transport (Fig. 9d). This suggests that the early phases of astrocyte remodeling involving increased connectivity may be a component of an endogenously protective mechanism, wherein these alterations within astrocytes positively influence the health of axons within the optic nerve. In late stages of neurodegeneration Cx43-mediated coupling helps disperse inflammatory cytokines through gap junctions [10, 15]. The data presented herein leads us to believe that Cx43 exhibits a multitude of functions, like astrocytes themselves. In these early stages of pathology, gap junctions allow astrocytes to couple with neighboring cells and share signals that may contribute to the redistribution of resources to those regions most at risk.

## Conclusions

Early optic nerve pathology involves astrocyte process remodeling, first to a higher state of organization and gap-junction coupling and then to gradual deterioration of organization as coverage increases and axons are lost. Morphologically, therefore, astrocytes exhibit multiple phases of remodeling due to neurodegenerative stress. We find that these early astrocyte responses to neurodegeneration are associated with beneficial functional outcomes for neurons. This responsiveness includes elevation in the gap-junction protein Cx43. Together, these data provide evidence for an endogenously protective mechanism wherein astrocytes increase connectivity to positively influence optic nerve axonal health during neurodegenerative stress.
